# Coordination-Polymer-Derived Cu-CoO/C Nanocomposite Used in Fenton-like Reaction to Achieve Efficient Degradation of Organic Compounds

**DOI:** 10.3390/nano14020132

**Published:** 2024-01-05

**Authors:** Linxu Xu, Rupeng Liu, Yubo Zhao, Xue Shen, Cuizhen Sun, Zhigang Yang, Jin Wang, Yufeng Du, Shuying Geng, Feiyong Chen

**Affiliations:** 1 Institute of Resources and Environment Innovation, Shandong Jianzhu University, Jinan 250101, China; xulinxu20@sdjzu.edu.cn (L.X.); zhaoyubo21@sdjzu.edu.cn (Y.Z.); shenxue20@sdjzu.edu.cn (X.S.); yangzhigang20@sdjzu.edu.cn (Z.Y.); wangjin21@sdjzu.edu.cn (J.W.); duyufeng22@sdjzu.edu.cn (Y.D.); gengshuying22@sdjzu.edu.cn (S.G.); 2 School of Municipal and Environmental Engineering, Shandong Jianzhu University, Jinan 250101, China; sczh2901@126.com; 3 Jianda Ecological Environment Innovation Center, Shandong Jianzhu University, Huzhou 313000, China

**Keywords:** Fenton-like reaction, catalyst, copper, cobaltous oxide, carbon

## Abstract

In this paper, carbon-matrix-supported copper (Cu) and cobaltous oxide (CoO) nanoparticles were obtained by using coordination polymers (CPs) as a precursor. The aqueous solutions of copper methacrylate (CuMA) and cobalt methacrylate (CoMA) were preferentially prepared, which were then mixed with anhydrous ethanol to fabricate dual metal ion coordination polymers (CuMA/CoMA). After calcination under an argon atmosphere, the Cu-CoO/C nanocomposite was obtained. Scanning electron microscope (SEM) and transmission electron microscope (TEM) showed that the material has banded morphology, and the dual functional nanoparticles were highly dispersed in the carbon matrix. The prepared material was used in a heterogeneous Fenton-like reaction, with the aim of replacing traditional ferric catalysts to solve pH constraints and the mass production of ferric slime. The obtained nanocomposite showed excellent catalytic performance on the degradation of methylene blue (MB) at near-neutral conditions; the discoloration efficiency is about 98.5% within 50 min in the presence of 0.15 mmol/mL H_2_O_2_ and 0.5 mg/mL catalyst. And good reusability was verified via eight cycles. The plausible pathway for MB discoloration and the possible catalytic mechanism was also proposed.

## 1. Introduction

Fenton reaction (Fe^2+^/H_2_O_2_) is one of the widely used advanced oxidation reactions in the field of water treatment due to its simplicity and low cost. However, the traditional Fenton process requires strict acidic conditions to obtain high catalytic activity, and the generation and disposal of large quantities of iron sludge must be considered in engineering applications [1,2], which always cause additional environmental and economic concerns. More and more researchers have clearly realized that the development of a heterogeneous Fenton-like reaction system based on the activation mechanism of the traditional Fenton reaction process is the key to solving the above problem. Fe-based catalysts, such as Fe^0^, Fe_2_O_3_, Fe_3_O_4_ and α-FeOOH, are preferentially applied in a Fenton-like reaction [3,4,5]. Despite the fact that the above types of catalyst can effectively reduce the production of iron sludge, but are limited by the lower rate constant (0.001–0.01 m^−1^ s^−1^) at which H_2_O_2_ reduces Fe(III)/Fe^3+^ to Fe(II)/Fe^2+^, the catalytic efficiency of Fe-based catalysts is not satisfactory [6,7,8]. Thus, a variety of modification methods, such as the introduction of an electron-rich system and doping semiconductor metal particles, are used to increase the rate of electronic transmission [9,10]. It is undeniable that modified Fe-based catalysts not only show higher catalytic efficiency, but also exhibit better stability. For example, the Fe@Fe_2_O_3_/H_2_O_2_ system assisted by ascorbic acid is nearly 53 times better than the homogeneous Fe(II)/H_2_O_2_ system [11]. However, pH optimization remains a major defect of the Fe-based process. Furthermore, some studies show that iron ions form stable complexes with intermediates [12], which means that the active center of iron-containing catalyst is easy to be “trapped” and consequently it is possible to be “deactivated” in the face of complex organic pollutants. Although other studies have shown that adding specific complexing agents, such as humus or polypates, can stabilize iron ions to maintain better catalytic activity, this is only applicable to high-alkaline conditions [13]. In contrast, physical field-assisted catalytic processes face high energy input and have no potential to large-scale engineering application at present.

Developing new multiphase catalysts to replace iron-containing catalysts is considered an effective solution. Based on the mechanism that Fe^3+^/Fe^2+^ is reoxidized by H_2_O_2_ to produce active free radicals in the traditional Fenton process, researchers believe that transition metal elements with multi-electron valence states can theoretically activate H_2_O_2_. Some reports have confirmed that Cu, Co, Mn, Ni, Zn, Ce and their oxides show varying degrees of activation ability [14,15,16,17,18,19]. Among them, copper is considered to be a good candidate to replace Fe-based catalysts due to three reasons: (i) copper has similar redox peculiarity to iron; (ii) copper does not bind to intermediates, making it possible to promote complete mineralization of organic pollutants; and (iii) Cu^2+^/Cu^+^ has a higher conversion coefficient, allowing for an enhancement in catalytic efficiency [20]. Copper ions (Cu^2+^ and Cu^+^) were first used to activate H_2_O_2_, in which the generally accepted theory is that the reduction of Cu^2+^ by H_2_O_2_ is the rate-determining step [21], which also provides ideas for the use and development of copper-based catalysts. Accordingly, Cu_2_O is directly used in a heterogeneous Fenton-like reaction, showing high catalytic activity [22,23]. But the Cu_2_O system seems to be unsuitable for Fenton reaction process because of the instability of crystal structure. While for Cu-based solid catalysts (Cu, CuO nanoparticles), the activity is much lower than Cu^2+^ ions, with one major reason being that molecular oxygen will interfere with the redox cycle of copper [24]. Some researchers are committed to combining copper species and other metals to build composite materials to overcome these problems, such as γ-Al_2_O_3_ [25], iron oxides [26], rare metals [27], etc.; or developing ternary polyphase composites to form dual electronic centers, such as CuFe/CuFeO_2_ [28]; or increase the specific surface area via the introduction of inorganic materials to reduce molecular oxygen interference, such as mesoporous silica [29], carbon materials [30], C_3_N_4_ [31], etc. The above measures not only improve the catalytic activity of the copper-containing catalysts, but also strengthen their stability. In addition, single-atom catalysts have also been used in a Fenton-like reaction, showing excellent catalytic performance [32,33]. However, its economics and stability in the face of complex organic pollutants remain to be discussed.

Although the design and development of catalysts with composite structures can undoubtedly improve the catalytic efficiency, the authors believe that due to the particularity of the water treatment field, the solid catalysts with complex composition may be more likely to cause secondary pollution. Researchers often pay much attention to the modifications in copper-based catalysts, but ignore the microstructure, which is another important factor affecting the properties of materials. Furthermore, nanoparticles with smaller sizes tend to have better properties due to the higher surface atomic ratio. Our team is dedicated to the design and preparation of catalysts with a simple structure and high activity to achieve an efficient degradation of organic matter in water. The activity of the Cu-based catalyst is highly dependent on its material composition, structure and morphology. A suitable matrix that supports ultra-small sized and highly dispersed active species may merge the advantages of homogeneous and heterogeneous catalysis. The coordination polymer (CP) is a class of functional precursor for preparing functional materials with controllable morphology and size, and good chemical stability. We have reported a Pd/Co_3_O_4_ composite derived from CoMA /Pd(II) CP precursor which has an ordered structure based on XRD measurement [34]. Thus, in this paper, coordination polymers were preferentially designed to obtain a carbon matrix composite. Methacrylic acid (MA) was chosen as the organic ligand due to its considerable elemental carbon ratio and the strong coordination capacity of carboxyl groups. In addition to copper ions, cobalt ions were also selected as functional metal precursors taking into consideration the increasing requirement of reusability and sustainability. The corresponding stable aqueous solutions of metal methacrylate salt (CuMA·H_2_O and CoMA·H_2_O) were obtained via the coordination reaction between methacrylate anion (MA^−^) and metal ions. Then, anhydrous ethanol was added to the mixture of metal methacrylate salt solution, facilitating the coordination of metal ions and carboxylate ions to generate coordination polymers (CuMA/CoMA). It is worth noting that this was a spontaneous self-assembly process, which means the microstructure of CuMA/CoMA is long-range ordered and the dispersion of metal ions is at a molecular level, favoring the formation of ultra-high dispersed metal nanoparticles. After that, the carbon matrix was obtained via carbonization at a high temperature, and the functional metal ions were in situ formed in it.

## 2. Materials and Methods

### 2.1. Materials

Methacrylic acid (MA, ≥99%), cobalt carbonate (CoCO_3_, 99.9%) and basic cupric carbonate (Cu_2_(OH)_2_CO_3_, 99.9%) were purchased from Sigma-Aldrich (Shanghai, China); absolute ethanol (EtOH, ≥99.7%) and tert-Butanol (TBA, 99%) were purchased from Sinopharm Chemical Reagent Co., Ltd. (Shanghai, China); the water used in the experiments was deionized with the resistivity of 18.2 MΩ·cm^−1^. MA was distilled before use and other chemicals were used as received without further purification.

### 2.2. Preparation of Aqueous Solution of Metal Methacrylate Salts

MA (0.2 mol, 17 mL) was dissolved in 100 mL deionized water, and then CoCO_3_ (0.1 mol, 11.8 g) was slowly added into the MA solution under vigorous stirring; after 1 min, another 50 mL of deionized water was added into the above mixed solution. The reaction was carried out under 60 °C water baths for at least 24 h. After filtration, the filtrate was vacuum-distilled to remove unreacted MA, and then the remainder was collected to store at 8 °C (named as CoMA·H_2_O). CuMA·H_2_O was obtained in the same way.

### 2.3. Preparation of Cu-CoO/C Nanoribbon

CuMA·H_2_O (15 mL) and CoMA·H_2_O (20 mL) were mixed in a beaker and shaken for a while, and then 100 mL absolute ethanol was added into the beaker. The mixture remained stationary at 50 °C for at least 4 h after centrifugation and washed with ethanol 3 times. The coordination polymer CuMA/CoMA was obtained and dried using a vacuum oven for 24 h. Finally, the Cu-CoO/C nanoribbon was obtained via the calcined CuMA/CoMA precursor at 500 °C in argon.

### 2.4. Catalytic Performance

The methylene blue (MB) solution was used to simulate printing and dyeing wastewater to test the catalytic performance of the obtained material. Briefly, 0.5 mg·mL^−1^ Cu-CoO/C material was firstly dispersed into a 100 mL MB solution (0.01 mg·mL^−1^) for 20 min to reach adsorption equilibrium. After that, 100 mM 30% H_2_O_2_ was added into the reaction system. At certain intervals, 2.5 mL filtrate was collected for analysis; the change in the intensity at the maximum absorbance wavelength (λ_max_) of 664 nm was evaluated by using an Ultraviolet-visible spectrophotometer. In order to optimize the efficacy of this Fenton-like process, several operating parameters, including pH, amount of catalyst and oxidant dose, were explored and their corresponding effects on pollutant abatement are discussed. In the recycling study, the catalysts were magnetically separated from the solution. After washing with water twice, the catalyst was used in the next reaction run. The procedure was repeated 8 times.

### 2.5. Characterization

Fourier-transform infrared (FT-IR) spectra were recorded using a KBr carrier containing the powder sample by employing an ENSOR spectrometer. A JSM-7610Fplus scanning electron microscope (SEM) with a primary electron energy of 5 kV was employed to examine the surface morphologies of the products. TEM images were obtained by applying a FEI Tecnai G2F30 transmission electron microscope with an accelerating voltage of 200 kV. X-ray diffraction (XRD) data were collected with a Rigaku D/Max-2500 X-ray diffractometer using a Cu target radiation source. The Raman spectrum in the range of 500–2000 cm^−1^ was determined using a Raman spectrometer (HORIBA Scientific) with laser energy of 532 nm wavelength. Ultraviolet-visible (UV-Vis) spectra were acquired at room temperature using a SHIMADZU 3100 UV-Vis-near-IR spectrophotometer. X-ray photoelectron spectra (XPS) were collected using a VG ESCALAB MKII with Al Ka excitation (1361 eV). Binding energy calibration was based on the C 1s spectrum at 284.6 eV. The degradation intermediates of MB were analyzed using liquid chromatography–mass spectrometry (LC-MS, LTQ, Thermo Fisher Scientific, Waltham, MA, USA). All mass spectra were recorded in full scan mode over the range from 50 to 1000 *m*/*z* for qualitative analysis. Inductively coupled plasma (ICP) atomic emission spectroscopy measurements were performed on an Optima 7000 DV.

## 3. Results and Discussion

### 3.1. Characterization

**Scheme 1:**
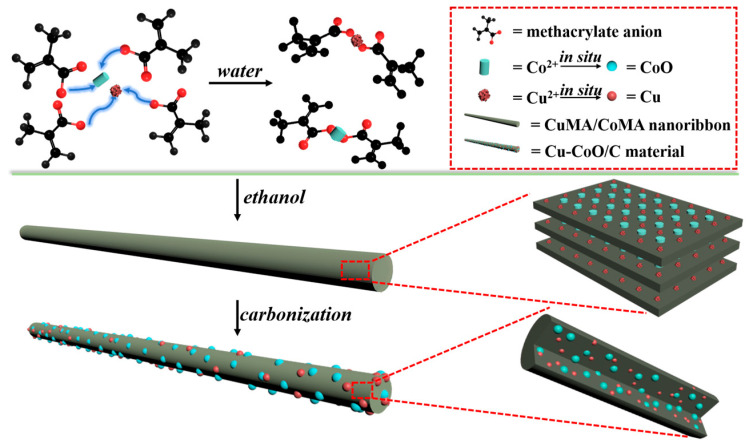
Synthetic pathway for preparing Cu-CoO/C nanoribbon.

The synthetic route of the Cu-CoO/C nanoribbon is shown in Scheme 1. MA is a moderate-strength organic acid with the pKa value of 4.66, which means it can react with most metal salts. Carboxylate is a very strong coordination group and can coordinate with almost all metals, while the amphiphilic methacrylate salt tends to form a coplanar conjugated structure. Firstly, CoCO_3_ and Cu_2_(OH)_2_CO_3_ were reacted with MA in water to obtain the solutions of CoMA·H_2_O and CuMA·H_2_O, respectively. Generally speaking, solvation is responsible for the formation of stable aqueous solutions, such as hydrogen bonding between the methacrylate anion and the water molecule, which allows the CoMA·H_2_O and CuMA·H_2_O solutions to remain stable for several months, but also prevents the binding between the methacrylate anion and the metal cations. Thus, the solvation effect should be weakened and the coordination interactions should be reinforced to facilitate the combination between methacrylate anion and the metallic cations. A simple and effective method is to add a large amount of poor solvent to the aqueous solution of methacrylate metal salts. For this purpose, we selected ethanol to be mixed with the aqueous solution of methacrylate metal salts. Since ethanol is a strong hydrogen bond acceptor [35], water molecules will preferentially form hydrogen bond with ethanol, which must reduce large amounts of water molecules coordinating with carboxylate ions and metal ions. Namely, the addition of anhydrous ethanol can not only change the solvation, but also affect the coulomb interaction of solute molecules, thereby facilitating the coordination equilibrium to form CuMA/CoMA assembled structures. Therefore, the resulting mixture was still for a certain time to grow CuMA/CoMA assemblies according to the desolvation mechanism. In fact, the addition of a poor solvent promotes the coordination of metal ions, and carboxylate is a very typical supramolecular self-assembly process. Figure S1 (Supporting Information) shows the FT-IR spectrum of the as-obtained CuMA/CoMA assembled material. A strong absorption peak presented at 1574 cm^−1^ can be assigned to the antisymmetric stretching vibration peak of carboxylate radical (COO^−^). Moreover, a classic acromion presented at 663–617 cm^−1^ gives further information of the twisting vibration of carboxylate radical (COO^−^). Since no characteristic peaks of carboxyl (-COOH) were presented, we believe that hybrid CuMA/CoMA was successfully synthesized.

**Figure 1:**
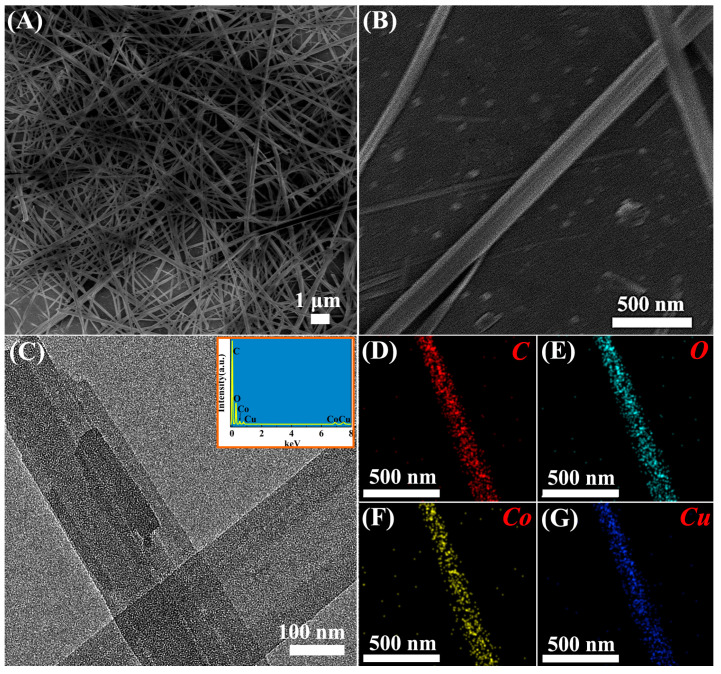
SEM images (**A**,**B**); TEM image (**C**); and EDX element mappings (**D**–**G**) of CuMA/CoMA nanoribbon (inset in (**C**) is the EDS spectrum).

The micro-structure of CuMA/CoMA assembled material was characterized via SEM and TME measurements. From Figure 1A, we can see that the as-obtained CuMA/CoMA assembled material has a ribbon morphology with the length of a range of dozens of microns. Figure 1B gives the information that the CuMA/CoMA nanoribbon has a width of ~200 nm, and the surface of this material is very smooth. Figure 1C shows the TEM image of the CuMA/CoMA nanoribbon, which can be observed to have a very uniform internal structure. The insert in Figure 1C shows the EDS spectrum of the CuMA/CoMA nanoribbon with information on the elements of Cu, Co, O and C. In order to explore the distribution of copper and cobalt elements, EDX element mapping was employed and the results are shown in Figure 1D–G, which shows that Cu and Co ions are homogeneously dispersed in the organic carbon matrix, allowing for the generation of carbon material loaded with highly dispersed active species.

**Figure 2:**
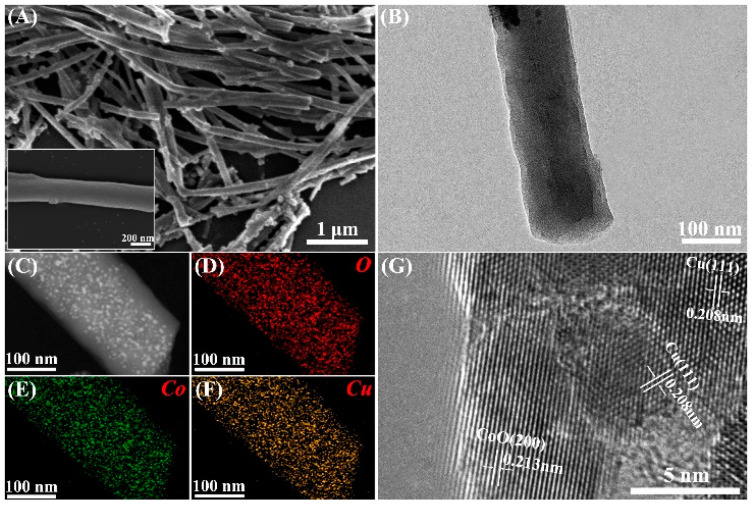
SEM image (**A**); TEM image (**B**); HAADF image (**C**); EDX element mappings (**D**–**F**); and HRTEM image (**G**) of Cu-CoO/C nanoribbon (inset in (**A**) is the selected enlarged SEM image).

Cu-CoO/C material was obtained after carbonization. In this process, a carbon skeleton transformed into a carbon matrix and metal ions were in situ generated. The structure of the Cu-CoO/C material is shown in Figure 2. From Figure 2A,B, we can see that the ribbon morphology was still maintained after carbonization, which illustrates the great mechanical strength of the assembled material. The insert enlarged image in Figure 2A also reveals the smooth surface of the carbonization material. Figure 2C shows the HAADF image of the Cu-CoO/C nanoribbon; two regions with different shades can be observed, representing the metal particles and the carbon substrate, respectively. EDX element mappings of O, Co and Cu are shown in Figure 2D–F, revealing the great dispersity of Cu and CoO nanoparticles. Figure 2G is the HRTEM image of the Cu-CoO/C nanoribbon; we can see that Cu and CoO nanoparticles with high crystallinity are separated from each other. The presented crystalline lattice with interplanar spacings of 0.208 nm and 0.213 nm corresponds to the (111) and (200) lattices of Cu and CoO nanoparticles (JCPDS No. 04-0836 and No. 43-1004). In addition, the mass fraction of Cu and CoO was employed via inductively coupled plasma (ICP) atomic emission spectroscopy, and the values are 12.5% and 14.8%, respectively.

**Figure 3:**
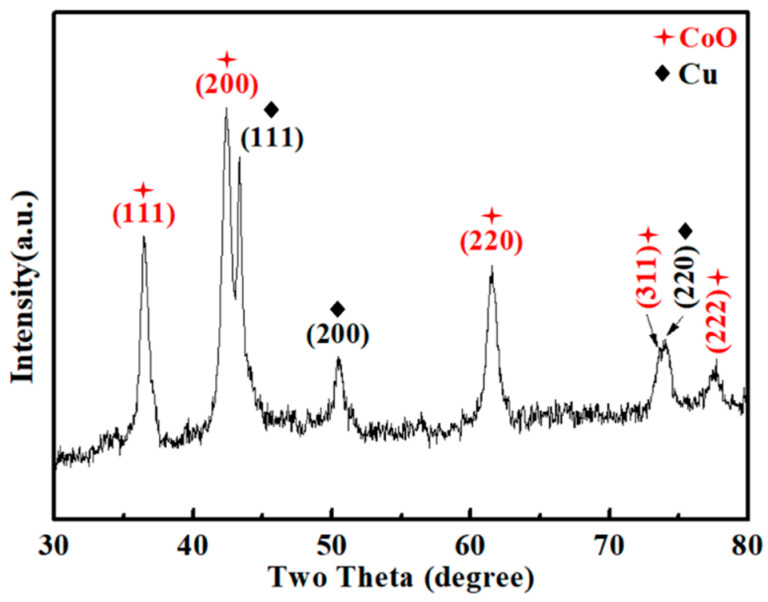
XRD pattern of Cu-CoO/C nanoribbon.

More detailed information about the crystal phases of the Cu-CoO/C nanoribbon was acquired from the XRD measurement. The results shown in Figure 3 demonstrate that there are two characteristic diffraction peaks. The ones marked with red stars presented at 2θ = 36.5, 42.4, 61.5, 73.6 and 77.6°, consistent with (111), (200), (220), (311) and (222) planes, are ascribed to the cubic face-centered phase CoO with cell parameters a = 4.26 Å (JCPDS No. 43-1004). The peaks marked with black diamonds located at 2θ = 43.3, 61.5 and 73.9° can be indexed to the (111), (200) and (220) planes of the cubic face-centered phase Cu nanoparticle, with the cell parameters a = 3.615 Å (JCPDS, No. 04-0836). The result of the XRD pattern demonstrates that both Cu and CoO with high crystallinity were in situ generated in the carbonization process, which is also consistent with the TEM tests.

**Figure 4:**
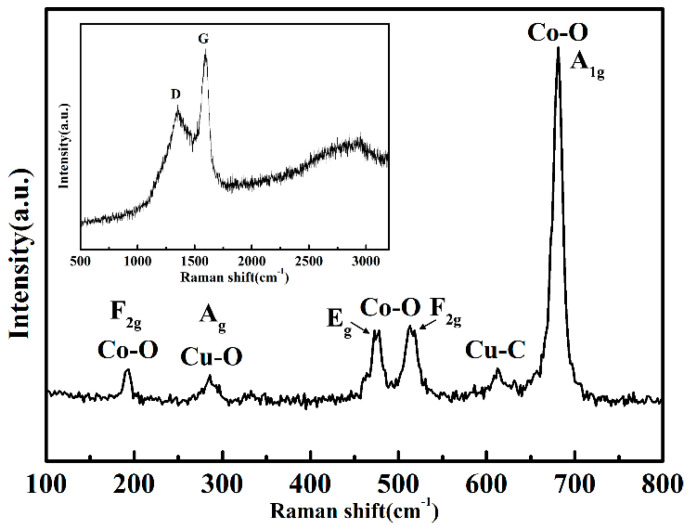
Raman spectra of Cu-CoO/C nanoribbon.

Figure 4 shows the Raman spectra of the Cu-CoO/C nanoribbon. The peaks presented at 192, 473, 515 and 680 cm^−1^ are in accordance with the F_2g_, E_g_, F_2g_ and A_1g_ modes of the CoO nanoparticle. The peaks at 285 and 613 cm^−1^ are assigned to the Cu-O and Cu-C bond, respectively [36]. The appearance of these two characteristic peaks can well indicate the existence of copper species, but we cannot verify the chemical composition of copper species based on this. The reason is that homojunction metals have no Raman signal. The XRD pattern confirmed the existence of elemental copper; so, the appearance of Cu-O bonds and Cu-C bonds revealed via Raman spectroscopy may be partially formed by copper ions with molecular oxygen and molecular carbon during carbonization. Moreover, it is worth noting that the Raman characteristic peak on the A_g_ mode of bulk CuO should appear at 297 cm^−1^. The reason for the downshift is that the nano-scaled particle has a large effect on Raman peaks due to the rules of crystal momentum conservation, which allows the phonon with wave vector to participate in the first-order Raman scattering; thus, phonon dispersion near the zone center must be taken into consideration [37]. As a result, the Raman peaks will downshift as the particle size decreases. The inset in Figure 4 presents the Raman spectra of the carbon matrix of the Cu-CoO/C nanoribbon, in which two remarkable peaks can be observed at about 1350 and 1590 cm^−1^. The two peaks are attributed to the D band and G band of carbon material, representing the defects and disorder of local sp^3^ and the E_2g_ mode of sp^2^ carbon atoms of graphite. The intensity ratio of D band to G band (ID/IG) can measure the surface defects of carbon material. It can be seen that the ID/IG value of this obtained material is about 0.73, suggesting higher graphitization of the Cu-CoO/C nanoribbon. Some research work has shown that the Raman characteristic peak of multilayer graphene, called 2D band, appears at about 2700 cm^−1^. Apparently, there is a diffused peak at 2700 cm^−1^ in the insert Raman spectra; thus, we also attempt to verify whether we obtain multilayer graphene material or not.

**Figure 5:**
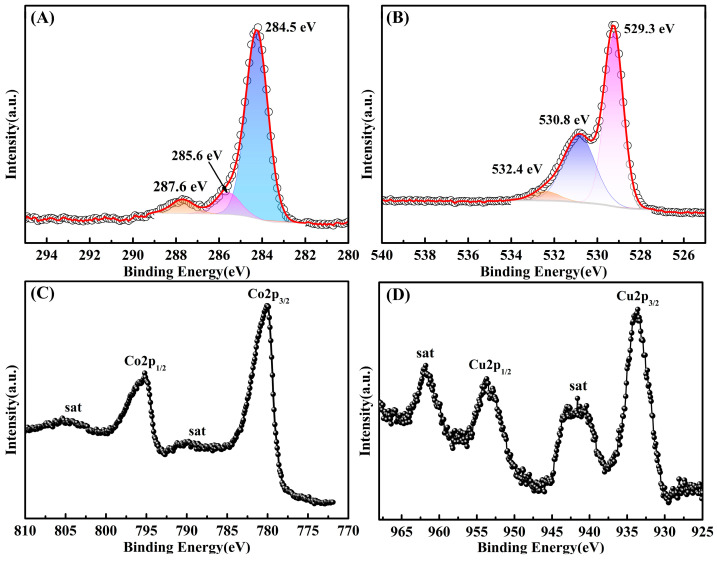
XPS spectra of Cu-CoO/C nanoribbon: fitting spectra of C 1s (**A**) and O 1s (**B**); Co 2p spectrum (**C**) and Cu 2p spectrum (**D**).

The elemental composition and valence state of metal species were measured via XPS. The C1s spectrum shown in Figure 5A was fitted into three deconvolution peaks, from which we can see that the main peak is located at 284.5 eV, revealing the C-C bond of defect-free graphite [38]. Another two deconvolution peaks located at 285.6 and 287.6 eV are attributed to defect carbon and the C=O bond [39]. The C1s spectrum also reveals high graphitization of carbon matrix, which is consistent with the Raman test. Figure 5B shows the XPS spectrum of O1s of the Cu-CoO/C nanoribbon; there are three deconvolution peaks presented at 529.3, 530.8 and 532.4 eV, which correspond to the lattice oxygen, the oxygen vacancies and chemisorbed oxygen [40]. The percentages of the three oxygen species are about 62.23%, 35.35% and 2.41%. Abundant oxygen vacancies can optimize the adsorption energy of reactants on the catalyst surface, thereby reducing the reaction energy barrier and promoting molecular activation. Figure 5C shows the XPS spectrum of Co2p, with two main peaks presented at binding energies of 780.1 eV for Co2p_3/2_ and 795.6 eV for Co2p_1/2_ (the difference value is 15.5 eV), indicating the characteristic of the CoO species [41]. The Cu2p spectrum is shown in Figure 5D; the two spin–orbit doublets located at 933.7 and 953.7 eV are assigned to Cu2p_3/2_ and Cu2p_1/2_, as well as the two satellite peaks with binding energies of 942 and 962 eV, indicating the characteristic of the CuO species [42], which suggested the oxidation of Cu(0) on the particle surface.

### 3.2. Effect of Catalyst Dosage

**Figure 6:**
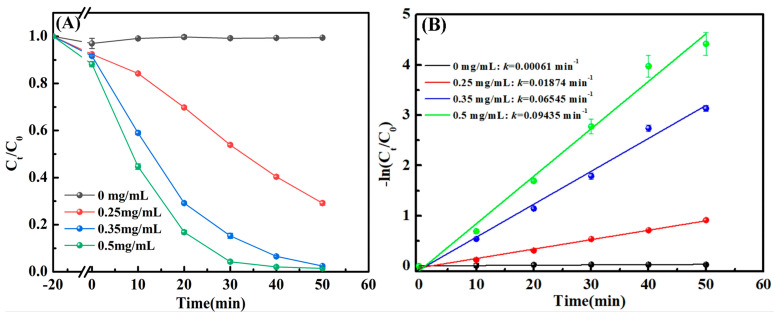
MB degradation curves under different dosages of catalyst (**A**) and the rate constant (k) estimated using the slopes of the straight lines of −ln(C_t_/C_0_) vs. reaction time (**B**) (Experimental conditions: MB 0.01 mg·mL^−1^; H_2_O_2_ 0.15 mmol/mL; pH = 7 and T = 298 K).

The synthesized Cu-CoO/C nanoribbon was applied to Fenton-like degradation of the MB dye as a heterogeneous catalyst. The catalytic performance of this sample was initially studied with the dosage of the catalyst. As shown in Figure 6A, MB concentration almost has no attenuation in the absence of catalyst, from which can be concluded that pure H_2_O_2_ has a poor effect on MB degradation. But, obviously, the discoloration rate of MB was drastically promoted by adding the catalyst. With an increase in the Cu-CoO/C nanoribbon from 0.25 mg/mL to 0.5 mg/mL, the discoloration rate increased from 70.8% to 98.5% within 50 min. This indicates that the prepared material can promote H_2_O_2_ to produce reactive oxygen species (ROS). Figure 6B shows the rate constant of MB discoloration at a different catalyst dosage; the pseudo-first-order kinetic fitting results showed that the reaction rate constant at 0, 0.25, 0.35 and 0.5 mg/mL catalyst is 0.00061, 0.01874, 0.06545 and 0.09435 min^−1^, respectively. The reaction rate is increased by 154.7 times with increasing catalyst dosage from 0 to 0.5 mg/mL, which indicated that the Cu-CoO/C nanoribbon exhibits excellent catalytic activity for promoting H_2_O_2_ to generate ROS.

### 3.3. Effect of H_2_O_2_ Dosage

**Figure 7:**
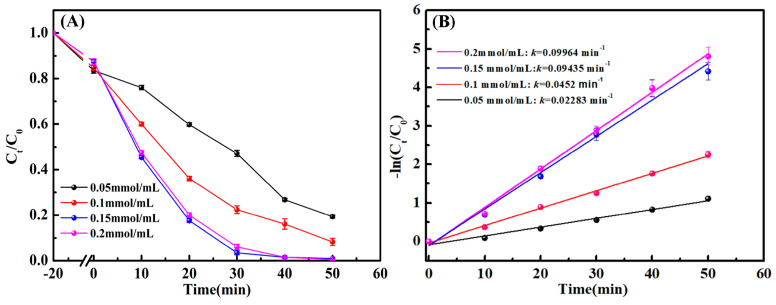
MB degradation curves under different dosages of H_2_O_2_ (**A**) and the rate constant (k) estimated using the slopes of the straight lines of −ln(C_t_/C_0_) vs. reaction time (**B**) (Experimental conditions: MB 0.01 mg·mL^−1^; catalyst 0.5 mg/mL; pH = 7 and T = 298 K).

The effect of H_2_O_2_ on discoloration MB is shown in Figure 7. In Figure 7A, we can see that MB discoloration is enhanced with increasing H_2_O_2_ from 0.05 mmol/mL to 0.2 mmol/mL. Actually, MB discoloration has already reached up to 80.7% in the presence of 0.05 mmol/mL H_2_O_2_ within 50 min, further indicating the excellent catalytic performance of the Cu-CoO/C nanoribbon. When the amount of H_2_O_2_ increased to 0.15 mmol/mL, the discoloration rate of MB reaches 98.5% after 50 min of catalysis with Cu-CoO/C nanoribbon. But with an increase in H_2_O_2_ from 0.15 mmol/mL to 0.2 mmol/mL, the discoloration rate is slightly promoted, which indicates excessive H_2_O_2_ is not necessary for Fenton-like process. Figure 7B shows the rate constant of MB discoloration with a different usage of oxidant; this process agrees with pseudo-first-order kinetic fitting and the results show that the reaction rate constant at 0.05, 0.1, 0.15 and 0.2 mmol/mL H_2_O_2_ is 0.02283, 0.04520, 0.09435 and 0.09964 min^−1^, respectively.

### 3.4. Effect of pH Value

**Figure 8:**
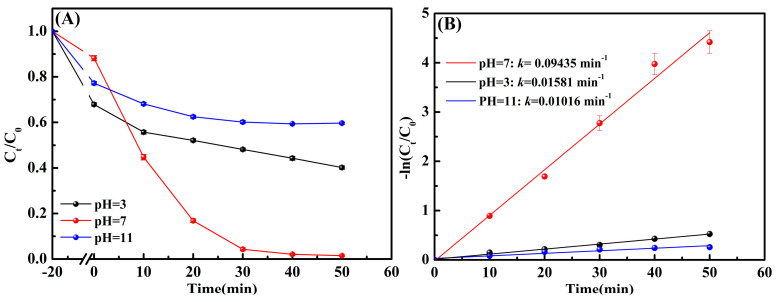
MB degradation curves under different pH values (**A**) and the rate constant (k) estimated using the slopes of the straight lines of −ln(C_t_/C_0_) vs. reaction time (**B**) (experimental conditions: MB 0.01 mg·mL^−1^; catalyst 0.5 mg/mL; H_2_O_2_ 0.15 mmol/mL and T = 298 K).

The pH value is an important factor in a heterogeneous Fenton-like reaction, as it has a great effect on the stability of the oxidant and activity of the catalyst. As shown in Figure 8A, the discoloration of MB is most effective at pH = 7, while in both strong acidic and alkaline environments, it was unsatisfactory. Under acidic conditions, H_2_O_2_ and H^+^ ions tend to form stable [H_3_O_2_]^+^, which tremendously hinder the decomposition of H_2_O_2_ into ROS [43]. That is why it shows relatively a poor discoloration of MB. Under mild neutral conditions, H_2_O_2_ can be well activated, resulting in considerable ROS. Increasing the pH from 7 to 11 leads to the decrease in MB decomposition; the reason might be the decline in oxidation potential of ROS and instability of H_2_O_2_ that can be self-decomposed to H_2_O and O_2_ at high pH conditions [44]. Figure 8B reveals the rate constant at different pH conditions; the k value at pH 7 is 17.2 times and 8.8 times that at pH 11 and pH 3, respectively.

The major drawback of the traditional Fenton process and the heterogeneous catalytic process developed from that is the requirement of strongly acidic conditions. The requirement of acidic conditions undoubtedly increases the process’ cost and reduces the stability of the catalyst. The catalyst we reported in this work can work continuously and stably under neutral conditions, which has great advantages for real application in Fenton-like reactions.

### 3.5. Reusability and Stability of Catalyst

**Figure 9:**
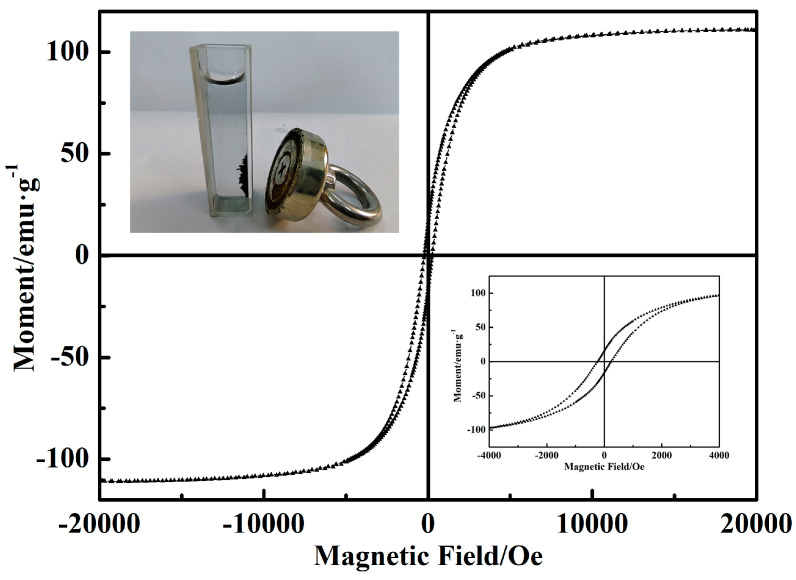
Magnetization curves at 295 K of Cu-CoO/C nanoribbon. The inset images are the digital image and the selected enlarged magnetization curves.

**Figure 10:**
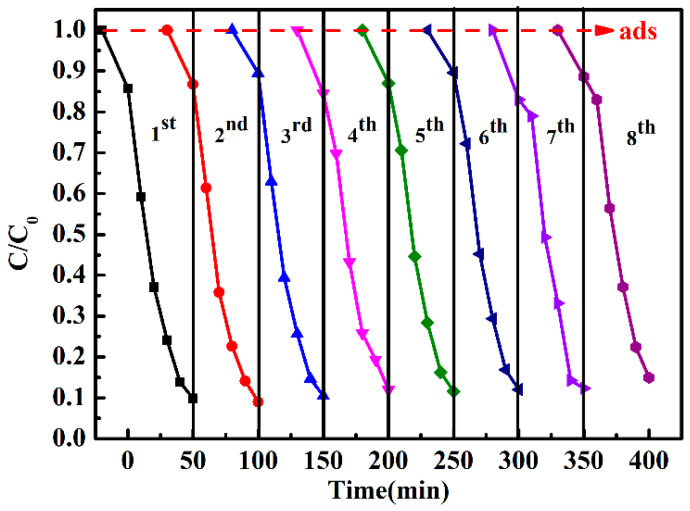
Cycling catalytic performance of Cu-CoO/C in the Fenton-like degradation of MB.

Good cyclic stability is another important index of catalyst. The CoO component endows the composited material with a magnetic property. Figure 9 shows the magnetization curves of the obtained catalyst; the value of saturation magnetization is 110.9 emu·g^−1^, and the obvious hysteresis loop suggests the ferromagnetic nature of the catalyst. An excellent magnetic property allows the catalyst to be easily separated from the reaction system and used for the next reaction (see inset digital image). We investigated the durability of the Cu-CoO/C nanoribbon under the same condition, and the result shown in Figure 10 indicates that the discoloration rate of MB could still reach more than 85% after eight cycles, suggesting good reusability of this obtained material. Moreover, the concentrations of metal ions leached from the Cu-CoO/C nanoribbon were also detected via ICP; the value of Cu^2+^ ions was 0.082 mg/L and 1.28 mg/L after one cycle and eight cycles, while that of Co^2+^ ions was 0.094 mg/L and 2.15 mg/L.

### 3.6. Possible Mechanism

In order to distinguish which components are dominant in Fenton-like reactions, some comparative experiments were conducted. The adsorption of MB was measured first and the result shown in Figure S2 (Supporting Information) indicates that MB adsorption is negligible under experimental conditions, probably because of the small surface area of the obtained material. Furthermore, we tested the catalytic performance of the carbon matrix after leaching metal particles based on the reports that the carbon material has the function of activating H_2_O_2_ for oxidation of organic pollutants [45]. The result (Figure S2) shows that the carbon in the Cu-CoO/C nanoribbon is insignificant for a catalytic degradation of MB dye. Moreover, the CoO/C material was synthesized and then catalytic discoloration of MB was performed in the same condition. The SEM image and XRD pattern shown in Figure S3 give the information of the obtained CoO/C material. The amount of CoO in the CoO/C material was measured via ICP and the value is 16.4%. The result of the discoloration of MB in the presence of the CoO/C nanoribbon shown in Figure S4 indicates that the highest discoloration rate of MB was about 39%, suggesting CoO has limited ability to activate H_2_O_2_. In order to further determine whether the Cu species play the key role in a Fenton-like system, the Cu/C material was synthesized and its catalytic performance was measured. The result of the discoloration of MB in the presence of Cu/C shown in Figure S5 indicates that the highest discoloration rate of MB was about 87.4%. Therefore, we concluded that the catalytic activity is majorly attributed to the Cu(0) species in the Cu-CoO/C nanoribbon.

**Figure 11:**
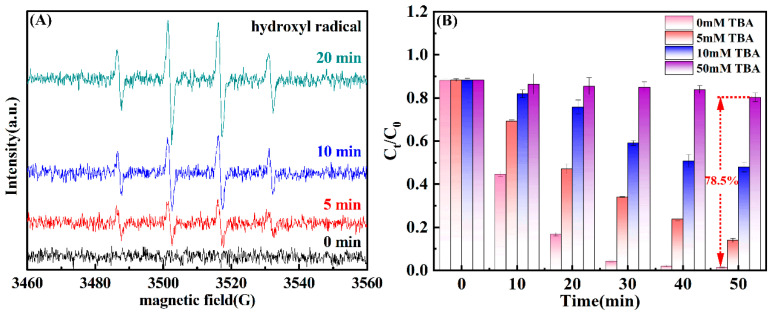
ESR spectra for the detection of ·OH/DMPO in the Fenton-like aqueous system catalyzed by Cu-CoO/C nanoribbon (**A**); and bar chart (**B**) of Cu-CoO/C catalyzing system by using n-butyl alcohol as ·OH trapping agent.

The ESR spin–trap technique was used to explore the oxidative species in the catalysis. As shown in Figure 11A, quartet peaks with a 1:2:2:1 intensity ratio that can be assigned to the signals of ·OH/DMPO adducts present in the Fenton-like system were catalyzed by the Cu-CoO/C nanoribbon, demonstrating the formation of ·OH species. Significantly, the concentration of generated ·OH is increased with the increase in reaction time, suggesting the catalyst can activate H_2_O_2_ continuously and quickly to achieve an efficient decolorization of organic dyes. The ·OH species are considered the dominant oxidant in a Fenton or Fenton-like system. A quenching experiment can effectively reveal the role of the ·OH species, and the result shown in Figure 11B indicates that TBA inhibits the discoloration of MB with an increase in the dosage of the scavenger. The MB discoloration was reduced by 78.5% in the presence of 50 mM TBA, which can directly verify the role of the ·OH species in a Fenton-like system catalyzed by the Cu-CoO/C nanoribbon.

**Figure 12:**
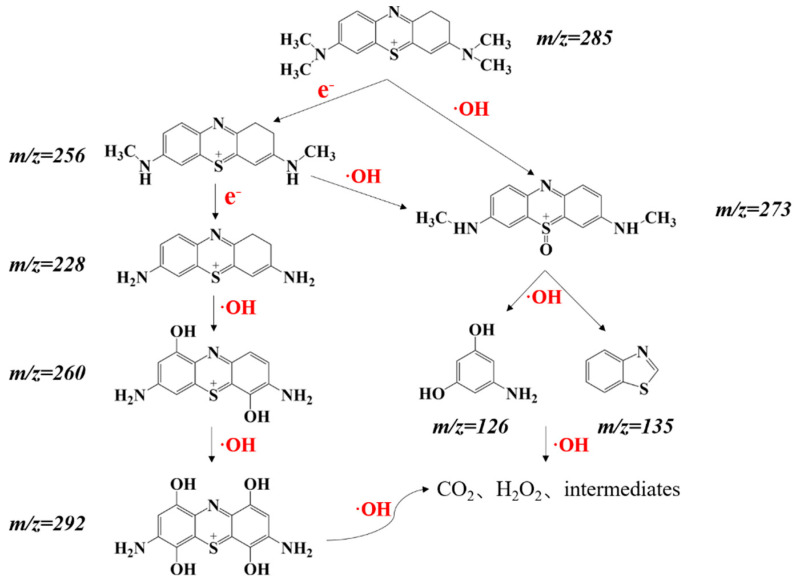
Possible pathway of MB discoloration.

LC-MS was used to explore the possible intermediates of MB discoloration. The result demonstrates that intermediates were mainly generated by demethylation and hydroxylation, which is similar to some reports [46]. The possible pathway of MB degradation is shown in Figure 12, and the peak at *m*/*z* = 285 was ascribed to the existence of MB molecule. According to one possible degradation route, the MB molecule is transformed into several fragments with *m*/*z* = 256 and 278 in the presence of electrons, namely demethylation. Peaks at *m*/*z* = 260 and 292 were detected, which can be attributed to the hydroxylation reaction after demethylation. In addition, ·OH is widely considered the main active species for organic degradation due to its extremely high oxidation potential; thus, another possible pathway is the direct interaction of ·OH with the MB molecule. The fragment of *m*/*z* = 273 was recorded, which should be caused by ·OH directly attacking the MB molecule. Additionally, small molecular rings with *m*/*z* = 126 and 135 were detected, illustrating the existence of the ring-opening reaction in the process of MB discoloration. Finally, all of the intermediates should be decomposed and mineralized into CO_2_, H_2_O, SO_4_^2−^ and NO^3−^.

**Scheme 2:**
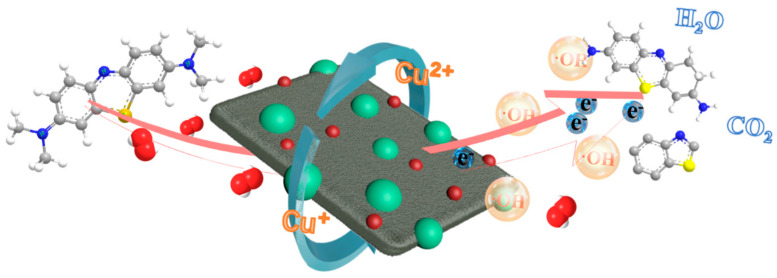
Proposed mechanism for the outstanding catalytic performance of Cu-CoO/C material in the Fenton-like degradation of MB dye.

Based on the above discussion, we proposed the possible mechanism of MB discoloration depicted in Scheme 2. The mechanism of heterogeneous catalysis is generally considered to be the process of “adsorption-catalysis-desorption”. In this Fenton-like system catalyzed by the Cu-CoO/C nanoribbon, the adsorption of MB and H_2_O_2_ on the surface of the catalyst is the beginning of the whole catalytic reaction. Afterwards, it is well accepted that H_2_O_2_ was activated to generate ·OH by a copper species (Equations (1)–(4)) [47]. After that, the ·OH and electrons with high oxidation activity can attack the MB molecule in an aqueous solution (Equation (5)).

Cu(0) + H_2_O_2_ + H^+^ → Cu^+^ + H_2_O (1)

Cu^+^ + H_2_O_2_ → Cu^2+^ + ·OH + HO^−^ (2)

Cu^2+^ + H_2_O_2_ → Cu^+^ + ·OOH + H^+^ (3)

Cu^2+^ + ·OOH → Cu^+^ + H^+^ + O_2_ (4)

OH + e^−^ → O_2_ + H_2_O + intermediates (5)

## 4. Conclusions

In this paper, we synthesized a Cu-CoO/C nanoribbon using the supramolecular assembly method. Both Cu(0) and CoO nanoparticles with ultrasmall size are highly dispersed in the carbon matrix. The Fenton-like reaction activities of the obtained material were explored via an MB discoloration experiment in an aqueous solution. The results showed that the Cu-CoO/C nanoribbon exhibited excellent catalytic performance under a near-neutral environment, and the discoloration efficiency is about 98.5% within 50 min in the presence of 0.15 mmol/mL H_2_O_2_ and 0.5 mg/mL catalyst. The Cu(0) species play the major role in activating H_2_O_2_ and the CoO component endows the material with excellent magnetic recovery function. In addition, this obtained material has good mechanical stability, and the discoloration rate of MB can still reach to 85% after eight cycles. The ·OH and high-energy electrons are considered the main active species in the MB discoloration reaction, and the possible degradation pathway was proposed as demethylation and hydroxylation. Given its low cost, abundant resources and easy preparation of copper species, we anticipate that the Cu-based catalyst can realize the deep oxidation treatment of organic wastewater.

## Data Availability

Data are contained within the article and Supplementary Materials.

## Supplementary Materials

The following supporting information can be downloaded at: https://www.mdpi.com/article/10.3390/nano14020132/s1, Figure S1: FT-IR spectrum of the obtained CuMA/CoMA nanoribbon; Figure S2: MB adsorption by Cu-CoO/C nanoribbon (a) and MB degradation in C/H_2_O_2_ system; Figure S3: SEM image and XRD pattern of CoO/C nanoribbon; Figure S4: MB degradation curves under different dosages of H_2_O_2_ (experimental conditions: MB 0.01 mg·mL^−1^; CoO/C 0.5 mg/mL; pH = 7 and T = 298 K); Figure S5: MB degradation curves under different dosages of H_2_O_2_ (experimental conditions: MB 0.01mg·mL^−1^; Cu/C 0.5 mg/mL; pH = 7 and T = 298 K); Figure S6: possible intermediates of MB discoloration detected via LC-MS. Figure S7–S9: full scan of UV-vis spectra of MB dye; Table S1: liquid chromatography parameters; Table S2: information of mass spectrum.
