# Supplementary material for: Coordination-Polymer-Derived Cu-CoO/C Nanocomposite Used in Fenton-like Reaction to Achieve Efficient Degradation of Organic Compounds
Source: Nanomaterials (Basel). 2024 Jan 5;14(2):132. doi: 10.3390/nano14020132 (PMC10819537; doi:10.3390/nano14020132)
Supplement: Supplementary file 1 [file nanomaterials-14-00132-s001.zip › nanomaterials-2739248-supplementary.pdf]

Supporting Materials

Coordination-Polymer-Derived Cu-CoO/C Nanocomposite Used in Fenton-like Reaction to Achieve Efficient Degradation of Organic Compounds

Linxu Xu [1], Rupeng Liu [1,*], Yubo Zhao [1], Xue Shen [1], Cuizhen Sun [2], Zhigang Yang [1], Jin Wang [1], Yufeng Du [1], Shuying Geng [1] and Feiyong Chen [1,3,*]

[1] Institute of Resources and Environment Innovation, Shandong Jianzhu University, Jinan 250101, China; xulinxu20@sdjzu.edu.cn (L.X.); zhaoyubo21@sdjzu.edu.cn (Y.Z.); shenxue20@sdjzu.edu.cn (X.S.); yangzhigang20@sdjzu.edu.cn (Z.Y.); wangjin21@sdjzu.edu.cn (J.W.); duyufeng22@sdjzu.edu.cn (Y.D.); gengshuying22@sdjzu.edu.cn (S.G.)

[2] School of Municipal and Environmental Engineering, Shandong Jianzhu University, Jinan 250101, China; sczh2901@126.com

[3] Jianda Ecological Environment Innovation Center, Shandong Jianzhu University, Huzhou 313000, China

* Correspondence: liurupeng@sdjzu.edu.cn (R.L.); chenfeiyong@sdjzu.edu.cn (F.C.)

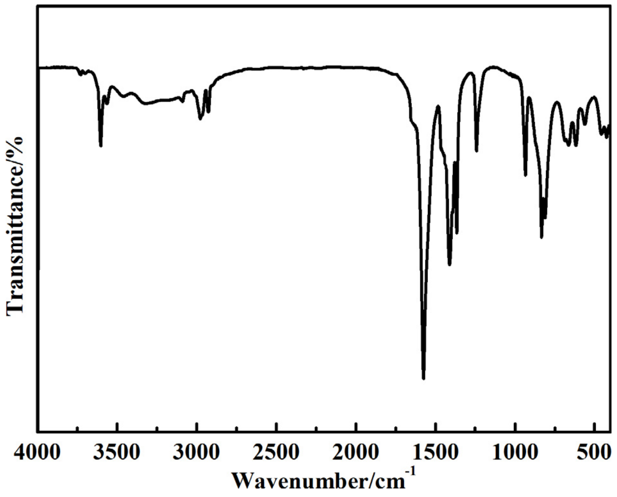

Figure S1. FT-IR spectrum of the obtained CuMA/CoMA nanoribbon.

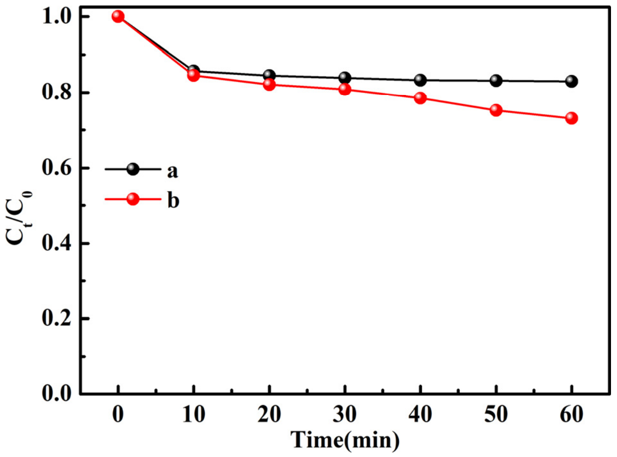

Figure S2. MB adsorption by Cu-CoO/C nanoribbon (a) and MB degradation in C/H_2O_2 system(b).

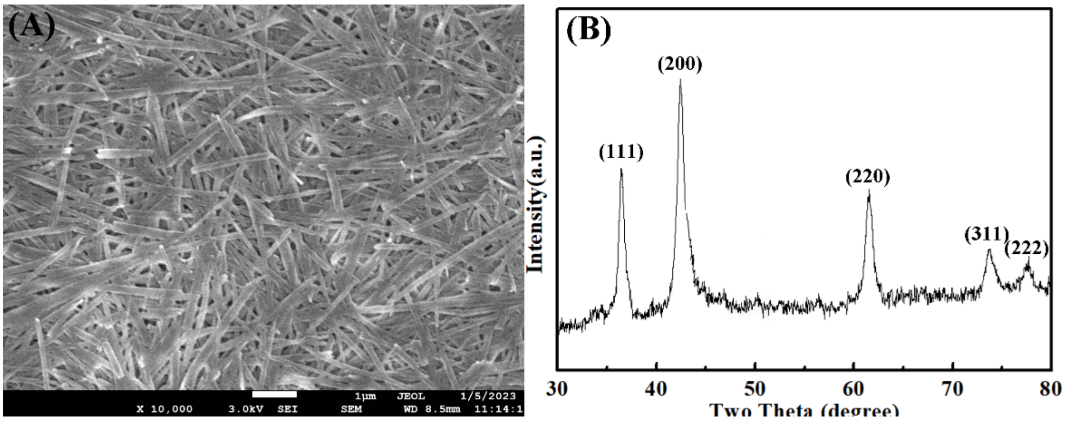

Figure S3. SEM image and XRD pattern of CoO/C nanoribbon.

SEM image shown in Figure S3(A) reveals the obtained CoO/C material has ribbon morphology. The XRD pattern is shown in Figure S3(B), the 2θ=36.5, 42.4, 61.5, 73.6 and 77.6°, consistent with (111), (200), (220), (311) and (222) planes, can be ascribed to the phase of CoO (JCPDS No. 43-1004). The amount of CoO in CoO/C was measured by inductively coupled plasma (ICP) atomic emission spectroscopy, and the value is 16.4%.

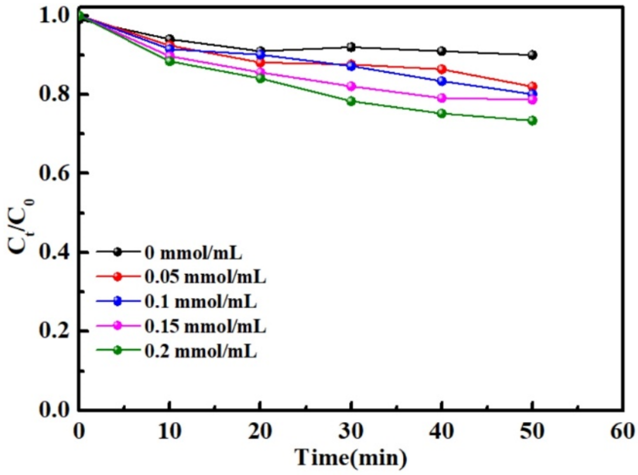

Figure S4. MB degradation curves under different dosages of H_2O_2 (Experimental conditions: MB 0.01 mg·mL^{-1}; CoO/C 0.5 mg/mL; pH=7 and T=298K).

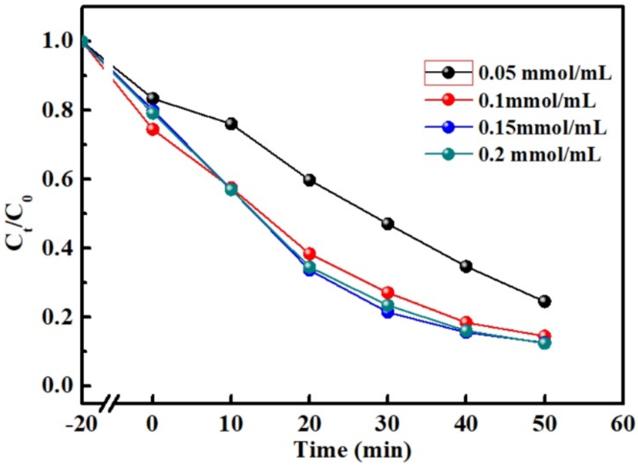

Figure S5. MB degradation curves under different dosages of H_2O_2 (Experimental conditions: MB 0.01 mg·mL^{-1}; Cu/C material 0.5 mg/mL; pH=7 and T=298K).

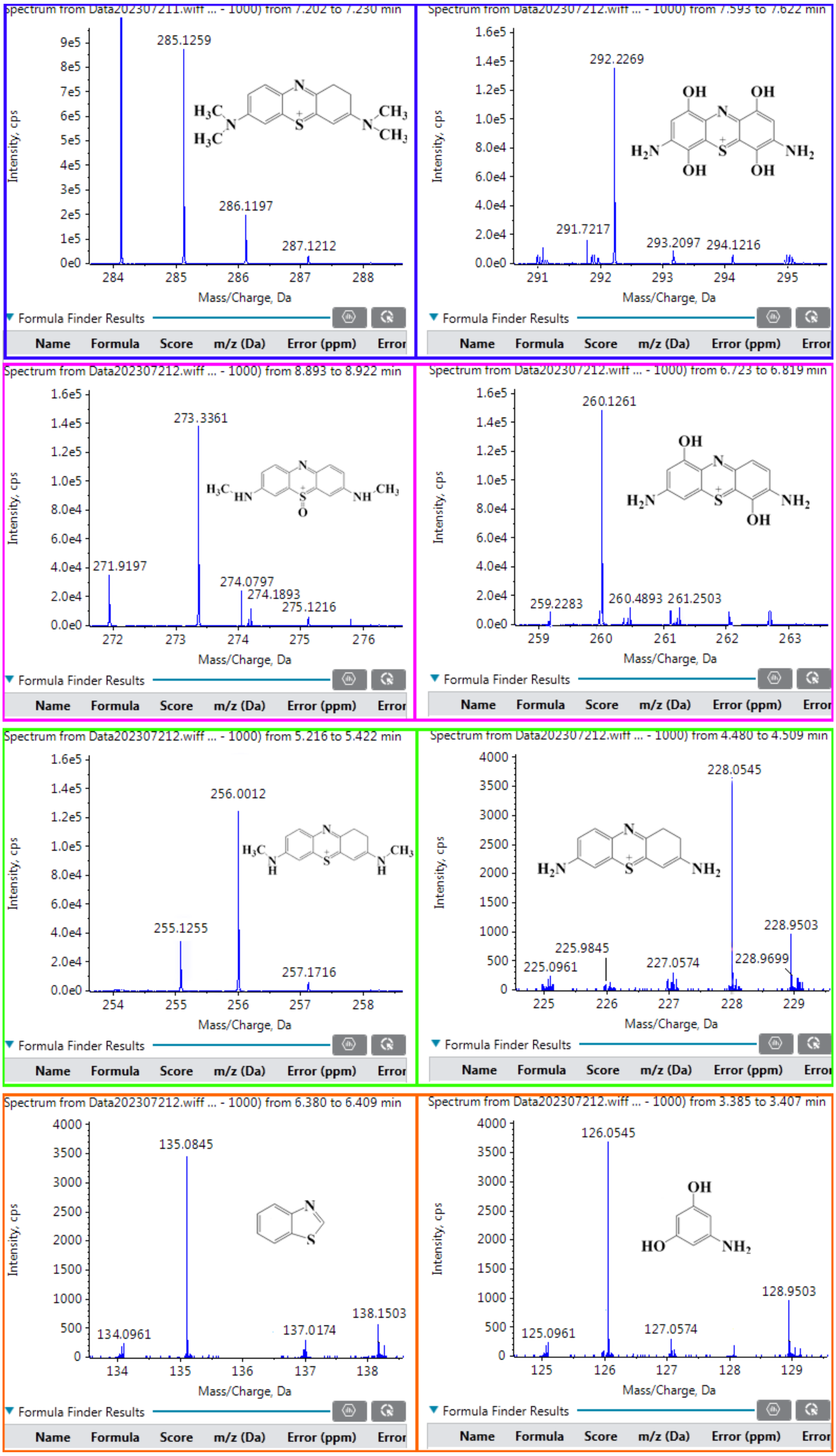

Figure S6. possible intermediates of MB discoloration detected by LC-MS.

Table S1. Liquid chromatography parameters.

Chromatographic column	Mobile phase	Sample size	Temperature of column	flow rate
Agilent RRHD-SB-C18, 2.1X50 mm, 1.8 um	A：acetonitrile<br>B：methane acid	5 μL	35℃	0.35 mL/min

Table S2. information of Mass spectrum.

Ion source	Model	Drying temperature	atomization pressure	scanned area	scanning time	Dryer flow rate	Capillary voltage
ESI	positive ion mode	150℃	30 Psi	50-1000m/z	300 ms	12L/min	3500V

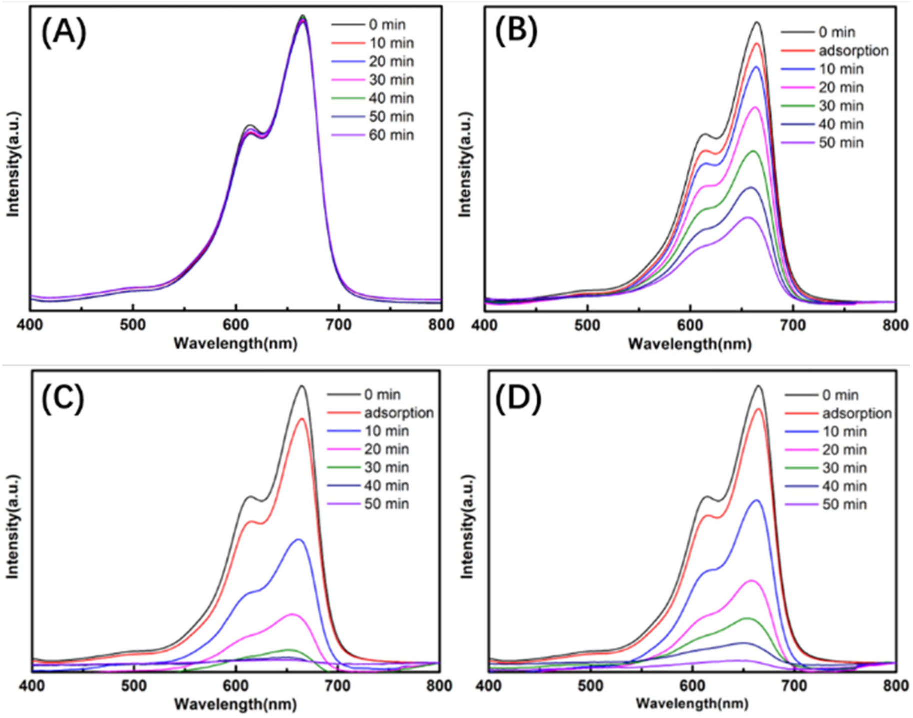

Figure S7. UV-vis spectra of MB dye in the presence of different dosages of catalyst: (A) 0 mg/mL; (B) 0.25 mg/mL; (C) 0.35 mg/mL and (D) 0.5 mg/mL (Experimental conditions: MB 0.01 mg·mL^{-1}; H_2O_2 0.15 mmol/mL; pH=7 and T=298K).

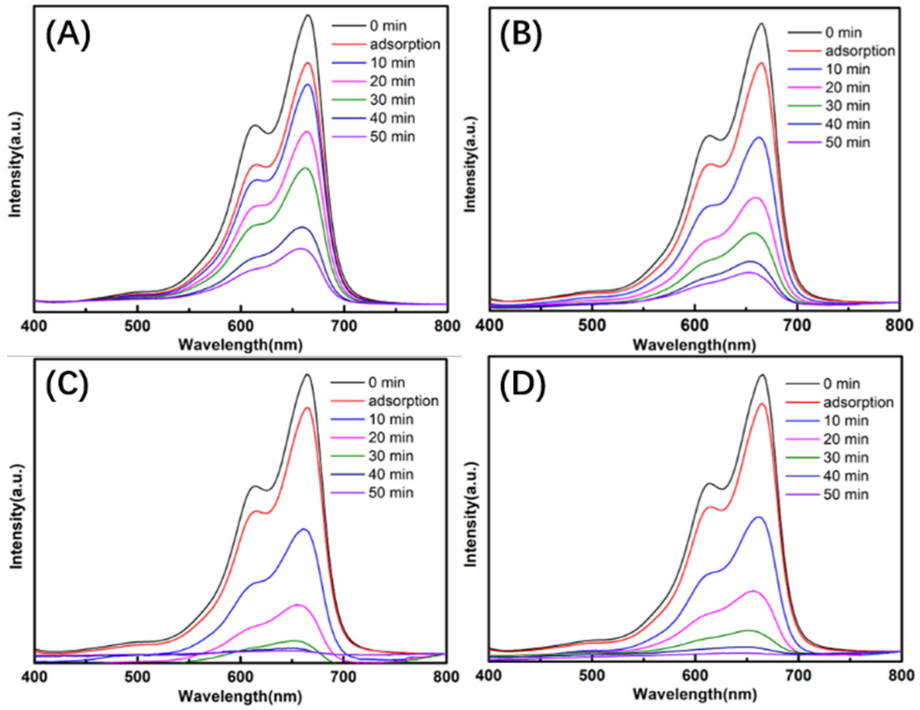

Figure S8. UV-vis spectra of MB dye in the presence of different dosages of H_2O_2: (A) 0.05 mmol/mL; (B) 0.1 mmol/mL; (C) 0.15 mmol/mL and (D) 0.2 mmol/mL (Experimental conditions: MB 0.01 mg·mL^{-1}; catalyst 0.5 mg/mL; pH=7 and T=298K).

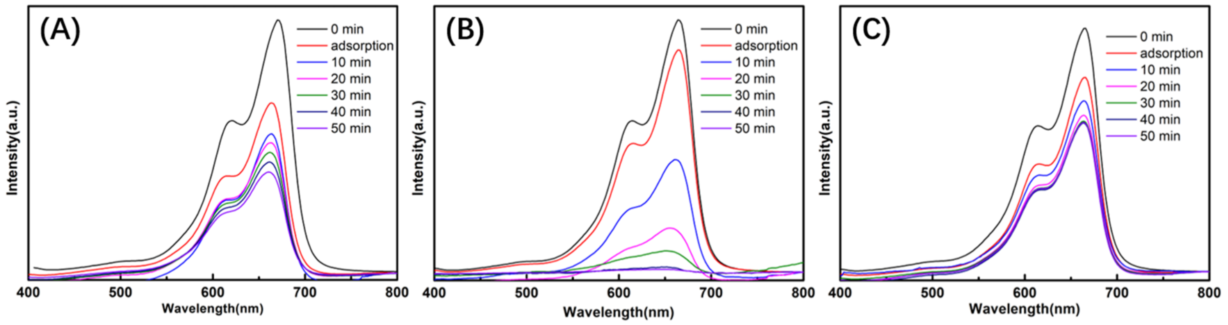

Figure S9. UV-vis spectra of MB dye under different pH conditions: (A) pH=3; (B) pH=7 and (C) pH=11 (Experimental conditions: MB 0.01 mg·mL^{-1}; catalyst 0.5 mg/mL; H_2O_2 0.15 mmol/mL and T=298K).
